# Prediction of 7 days mortality in a general population admitted to Emergency Department

**DOI:** 10.1186/1757-7241-20-S2-P40

**Published:** 2012-04-16

**Authors:** Charlotte Barfod, Jakob Lundager Forberg

**Affiliations:** 1 Department of Anaesthesiology, Hillerød Hospital, Denmark; 2 Department of Emergency Medicine, Hillerød Hospital, Denmark

## Background

Assessment and treatment of the acutely ill patient have improved by introducing systematic assessment at admission and accelerated protocols for specific patient groups. We have, however, very sparse knowledge of how the initial assessment and status of the general acutely ill patient admitted to Emergency Department is associated with patient outcome.

## Methods

The Hillerød Acute Process Triage (HAPT) is inspired by the Swedish Adaptive Process Triage (ADAPT) and ranks patients into 5 level colour-coded triage, ranging from red (most urgent) through orange, yellow, green and blue (least urgent). Patients are assigned a triage category for each of the to main descriptors 1) vital signs and 2) presenting complaint. The variable of the two associated with the most urgent triage category, will determine the final colour-coded triage category, which determines the level of patient observation and treatment. Data from admission including final triage category, vital signs triage category and presenting complaint triage category were prospectively registered and merged with the outcome measure: mortality within 7 days after admission. Patients in blue triage category were not admitted and therefore not included in this study.

## Results

A total of 6335 unique patient contacts were retrieved in the period September 22, 2009 to February 28, 2010. The distribution of patients in final triage categories was red; 4.4%, orange; 25.2%, yellow; 38.7% and green; 31.7%. The Odds Ratio for 7 days mortality was 14.7 (7.6-28.5, p<0.0001) for red patients, 3.8 ( 2.1-7.1, p<0.0001) for orange patients and 1.9 (1.0-3.6, p<0.05) for yellow patients compared to green triage group. In univariate regression analyses, the covariates: age, final triage category, vital sign triage category and presenting complaint triage category were all significantly associated with 7 days mortality (p<0.0001). These covariates were included in the subsequent multivariate analysis, leaving age and vital sign triage category as independent risk factors for 7 days mortality.

## Conclusion

HAPT is valid in terms of predicting short-term mortality in the general acute population. Age and triage category determined by admission vital signs are independent predictors of 7 days mortality. Including the presenting complaint in the triage model may result in over triage.

